# Book Reviews

**Published:** 1812-06

**Authors:** 


### CRITICAL ANALYSIS

### *OF RECENT PUBLICATIONS*

IN THE

### DIFFERENT BRANCHES OF PHYSIC, SURGERY, AND MEDICAL PHILOSOPHY.

*The Morbid Anatomy of the Human Gullet, Stomach, and Intestines. By* Alexander Monro, Jun. M.D. F.R.S.E. Professor of Medicine, Anatomy, and Surgery, in the University of Edinburgh, &c. &c. &c. Large 8vo, Edinburgh, 1811. pp. 592. xxi plates.

IN giving an analytical view of this large volume, the distribution of the subjects adopted by the author will be followed;* and though, perhaps, his arrangement is not the most perspicuous possible, yet, by treading in his footsteps, we shall present our readers with a more satisfactory account of his work, than could arise out of any attempted alteration of his plan, according to any notions of our own.

The Introduction, of xxv pages, is occupied with general observations on the advantages arising out of this branch of professional study; and in an elucidation of some particular points of importance. The peculiar advantages of the study of *morbid anatomy* the author enumerates under the five following heads.

"1st. This study, by elucidating the nature and progress of diseases connected with a derangement of organic structure, affords a

* "The arrangement adopted differs a little from that of preceding authors; for, instead of describing in continuance all the organic derangements of the gullet, of the stomach, or of the intestines, I have classed under one head the same diseases in their different parts of the alimentary canal, following rather the arrangement of diseases than parts." *Introd.* xix.

sure sure foundation upon which an opinion may be grounded, respecting the nature and progress of such diseases.

"2d. As the study proves that parts similar in their structure are subject to the same organic derangements, it exposes the fallacy of many hypotheses which have been propagated respecting organic diseases.

"3d. If parts of a similar structure be subject to similar organic derangements, *morbid anatomy* may tend to unfold the structure of many of the smaller parts of the human body, which have hitherto escaped observation, as it shews that many of these smaller parts are subject to the same organic derangements as those organs whose structure is obvious to our unassisted senses.

"4th. *Morbid*, like *comparative*, *anatomy*, assists in distinguishing those organs which are essential to life, from others of less importance, and also shews that every part, even of the same bowel, is not equally sensible or equally necessary to the prolongation of life.

"5th. This study points out the uncertainties and deficiencies of many parts of medical science, and particularly as to the method of cure; and hence may pave the way to a new and improved method of treatment."

These general observations on the advantages arising from examining and understanding the diseased alteration of structure in various parts of the animal frame, are illustrated by the particular examples of diseased liver, hernia, and stricture of the urethra.

"The pressure of the enlarged liver may interrupt the functions of the stomach; or the same cause, impeding the free circulation of the blood through the different bowels of the belly, as well as through the liver itself, may produce a dropsy of the belly. The enlarged liver is the source of still further mischief; by pressing upon the gall ducts, it impedes the free passage of the bile into the intestinal canal; and the bile thus obstructed, passes off by another channel, and is taken up by the lymphatic vessels, occasioning jaundice. Nor is this all the evil to be apprehended from the affection we are considering. By means of inflammation, the enlarged liver may be united with the neighboring parts; and if, in such a situation, an abscess should take place within, the contents of the abscess might be discharged into the general cavity of the belly, into the sacs of the pleuræ, or even into the lungs.

"The progress of the disease called rupture, or hernia, affords another and very striking example of the importance of the study of *morbid anatomy*; for even the most accurate knowledge of the anatomy of the body, in its sound state, conveys but an imperfect idea of the state of the displaced parts, or of the state of the canal through which the displacement has been propagated. The displaced portion of the intestine pushes before it the thin, slippery, and elastic, peritoneum, which lines the belly; and, after such an occurrence, the lining soon loses its natural characters, being converted verted into a dense, lamellated, membrane, which frequently contracts adhesions with the neighboring parts. The intestines, during the different stages of the disease, are more or less twisted, compressed, and often inflamed. By consequence of the displacement, their coats become thicker, as also the neighboring cellular substance.

"Of the preceding remarks, the disease called stricture of the urethra affords a very strong illustration. In the earlier stages of the (this) disease it only produces a membranous growth, which, in its future progress, is converted into ligament, and even into cartilage; and the neighboring parts are frequently reduced to the same morbid state. In addition to the pain and difficulty in discharging the urine, the usual concomitants of stricture, the disease often occasions various constitutional symptoms: among these may be enumerated great nervous irritation, despondency, in some cases bordering upon delirium, and, in other cases, symptoms similar to those which accompany an ague."

If the examination of diseased parts, *post mortem*, according to the rules of anatomical science, and with a reference to the symptoms during life, had needed a recommendation to those who feel the value and the advantage of collecting facts, Dr. Monro's Introduction would be particularly valuable; and, should there be any professional persons who still doubt the importance of this species of investigation, we refer them to the arguments here employed.

Before we proceed to a view of the contents of this volume, we shall venture a few cursory observations on the language employed in it, as well as in some other works, of the present period.

Morbid anatomy for the anatomy of diseased parts—Morbid poison for the contagious or infectious material generated in certain diseases—Female complaints for the diseases of the female sex—are expressions of doubtful import at best; and would always mislead without a previous pariphrastic explanation. This affected conversion of words, made, perhaps, with a view to compression, has done much toward destroying the perspicuity of the language of science. According to accepted usage, they express something very different from what is intended. Thus a female complaint is a disease of the feminine gender—morbid anatomy is the science itself in a state of disease. A thousand analogies will prove this to be the precise sense of the language: a gratuitous admission at first, may, in time and by custom, however, give to this phraseology, perhaps, a legitimate establishment. In the fluctuations of taste, many wilder innovations may arise; or we may return to the old standard.

"Multa renascentur, quæ jam cecidêre; cadentque,

Quæ nunc sunt in honore vocabula, si volet usus;

Quem penès arbitrium est, et jus, et norma loquendi."

Much

"Much phrase that now is dead, shall be reviv'd;
And much shall die, that now is nobly liv'd,
If custom please; at whose disposing will
The pow'r and rule of speaking resteth still."

The practice of others has usually been brought in excuse of employing what is incorrect in itself. Thus far Dr. Monro is supported in the title of his work; but we think it strange that a professor in a liberal and learned science, at one of the first schools in the world for teaching that science, should employ colloquial barbarisms in place of the language which the science itself furnishes, and which all *his* readers would better understand. Why should the disgusting term *gullet* be substituted for the naturalized œsophagus? The doctor apologises for his language on the ground of his being employed on subjects of more importance. Perhaps he is so employed; but he should be aware that neatness, perspicuity, and even elegance, of composition, are compatible with, and essential to, the success of his labors, when he presents those labors to the public. The language of his dissecting room, like his dissecting dress, may be negligent and coarse; but we should be hurt to descry the professor of medicine, anatomy, and surgery, issuing into the streets and public places enveloped in a greasy gown, and an old leathern travelling cap on his head. If the language of this volume is redundant without perspicuity, if familiar without ease, the author has injured medical literature by his example,

"The *better sort* should set before them
A grace, a manner, a decorum;
Something that gives their actions light,
Not only makes them great, but bright." *Prior.*

Having ventured thus far, *en passant*, on a subject we deem of some importance, especially in this age of invention and coinage, when new terms start up daily, to "fright the language from its propriety," we proceed to the body of the work.

The first Chapter consists of "General Observations upon the Structure and Morbid Anatomy of the Alimentary Canal," preliminary to the particular objects of the volume, which are classed under the six following heads.

1. Comprehends an explanation of the morbid effects which have resulted from hurtful substances swallowed by design or accident.

2. Organic affections peculiar to the coats of the alimentary canal.

3. The nature and distressing consequences of the displacement of a part of the alimentary canal.

 4. An

4. An explanation of the various mal-conformations of the alimentary canal.

5. Description of worms which occasionally infest the alimentary canal.

6. An enumeration of the causes which lead to an enlargement of those neighbouring organs, which, by pressing upon the alimentary canal, prove a mechanical obstruction to the progress of its contents.

Our readers will perceive the rich and interesting harvest which the subject promises, and expectation will not be disappointed.

"The greater part of the diseases of the alimentary canal, included in the above six classes, tend to obstruct the passage of the aliment to a greater or less degree; creating, in some instances, a permanent, in others only a temporary, obstruction.

"The permanent obstruction, by stricture, is of slow growth, but constant in its operation, and becomes greater and greater, either from the gradual increase of the concretion or tumor occasioning it, or from the gradual approximation of the opposite sides of the canal; whereas, the stricture from spasm comes on suddenly is generally of short duration, and the spasmodic contraction frequently goes off spontaneously, or after the exhibition of proper remedies.

"But upon some occasions the spasmodic contraction is not removed during life; and upon dissection we find the muscular coat of an unnatural hardness.

"There is in some constitutions a remarkable disposition to diseases which obstruct the *alimentary canal.* I have met with several patients who have been so unfortunate as to labor under two very different causes of obstruction at the same time, or in succession.

"The symptoms, originating from very different causes of obstruction, are in some respects similar; the patient suffers much from a severe tightness, pain, and soreness, in the diseased part.

"The mucous membrane being much irritated, the mucous, which is secreted in an extraordinary quantity, instead of being a mild fluid, becomes thin, of a light green or yellow color, and acquires an unnatural acrimony, so as sometimes to excoriate the neighbouring parts; and it is discharged in considerable quantity from the mouth, by hawking, or along with the fœces.

"Those mucous glands and their ducts, which are situated above the seat of the obstruction, being much irritated, attain an unnatural size, whilst those under it are not at all affected.

"The above symptoms are occasionally aggravated by catching cold*; and, in these circumstances, and from the irritable nature of the

* We would be glad to see the popular and vague term, *catching cold,* expelled from works of science. What does it strictly and truly the patient's constitution, an unnatural local spasmodic contraction is sometimes excited, which aggravates his sufferings.

"The constitution is at first but little affected, but, in consequence of the continuance of the obstructing cause, the digestive powers are much impaired. The patient loaths his food; what is taken becomes acid; he loses his spirits; and is occasionally very costive; but at other times much weakened by a bilious diarrhœa. He becomes weak and emaciated, and has a quick pulse: he suffers after a time very great pain, owing to the distention by air.

"The *air* accumulated in the stomach and intestines, sometimes distends these organs to such an uncommon size, that the convolutions of the intestines may be distinctly felt through the parieties of the abdomen.

"I have seen the stomach and intestines distended to two or three times their natural size, and sometimes even ruptured by the unnatural distention.

"It has been matter of dispute among physiologists, from what source the air is derived.

"It is not compatible with the object and limits of this volume, to enter at large into this question, which has afforded matter for so much controversy. I shall therefore only observe, that, as far as I can judge, a part of it is swallowed, a part is formed by secretion, and a part is generated by fermentation.

"As we sometimes meet with cases, where the distention, although so considerable as to occasion great pain and uneasiness, yet has gone off gradually, and without the discharge of air by the mouth or anus, it is probable that part of the accumulated air has been taken up by the absorbent vessels.

"From the abdomen being tense like a drum, where the stomach and intestines are distended by air; the name tympanites has been employed by nosologists, to express this morbid state.

"As the air sometimes escapes from the intestines into the general cavity of the abdomen, in consequence of wounds, ulceration, and other diseases, nosologists also describe the tympanites abdominalis.

"It is of some moment to distinguish the one species of tympany from the other, the former being a much less dangerous affection than the latter.

---

truely mean? Sometimes it meets us in the form of catarrh, or bronchitis; now it limps in rheumatism, suffocates in asthma, and our Professor employs it to aggravate the symptoms of obstruction in the intestinal canal. In short, it is the scape-goat of all our sins, with respect to the causes of disease. What is the cause of my complaint says the patient? The reply of the nurse, the apothecary, the surgeon, the physician, and the professor, is, you have *caught cold.* Thus have we gone on from age to age, but surely it is now time to question the validity of this catching cold. It may continue to be the shibboleth of old women, but the term should be discarded by men of sense and science.

"The abdominal tympany comes on suddenly, the belly is uniformly distended in whatever posture the patient is, and also smooth; the patient is not sensible of wind moving within his bowels, nor does he hear the sound of it; and he is not relieved by passing wind, nor is the distention of the belly diminished by a purgative.

"The intestinal tympany, on the other hand, comes on very gradually, the turns of the intestines may be traced by a careful examination; and the sound of the air, passing from the contracted to the dilated position, may be perceived.

"Besides, the patient is sensible of air moving from one place to another; and, whilst the air passes from one turn of the intestine to another, he suffers pain, the intestines being generally spasmodically straightened. The patient also discharges an usual quantity of air, upwards and downwards, by which he is much relieved.

"It may not be unnecessary to add, that the intestinal tympany has been mistaken for a dropsy of the belly; and, on the other hand, when a small quantity of water is contained within the cavity of the belly, and the intestines have at the same time been filled by air, the distended intestines float upon the water, and render it difficult, unless the patient be examined in different attitudes, to discover the water within the belly.

"An acute inflammation is sometimes the sequel of the immoderate distention of the stomach and intestines; it is rapidly communicated from one part of the abdomen to another, and very frequently proves fatal in a short time, by terminating in gangrene. But, in other cases, in the vicinity of the obstruction, we find the coats of that portion of the alimentary canal, immediately above the distended part, much thickened, in the same manner as the muscular structure of the heart and of the bladder of urine becomes much thickened, where there is an extraordinary impediment to the exit of the blood or urine.

"In such cases, the muscular fibres become thicker, redder, and stronger, to overcome the unnatural resistance.

"In some cases, the part immediately above the obstruction, is extended into a pouch. I have seen the gullet, when an extraneous body has been lodged in it for a considerable time, or when it has been obstructed by any other cause, dilated into a large pouch, which still farther obstructed the swallowing, and also the breathing; and, when that pouch is placed near to the termination of the gullet in the stomach, it may press so much upon the heart and lungs as to derange the functions of these organs.

"In a similar manner, the stomach, when the pylorus has been obstructed, sometimes becomes so prodigiously enlarged, as to reach the pelvis; and they are much dilated, from obstruction in any part of the intestinal canal. I have seen the intestinum rectum, in consequence of obstruction, so dilated, as to be capable of containing a child's head.

"The veins, in the vicinity of the obstruction, sometimes become enlarged, or varicose, and add materially to the bulk of the tumour; tumour; so that, when the rectum has been extended in consequence of an unnatural obstruction, it proves an impediment to the free discharge of urine, or the contents of the uterus.

"But the part beneath the seat of the obstruction undergoes a very opposite change; it is always somewhat contracted in its diameter.

"The unnatural pouch, created by the obstruction in the alimentary canal, generally adheres to the neighbouring parts. The distention continuing, some part of the pouch becomes thinner than the rest; the absorbent vessels are roused to act more powerfully than usual, by the stimulus of the unnatural distention; the sac becomes thinner and thinner, and at length bursts, or the unnatural sac is destroyed by ulceration; thus a communication is formed between the neighbouring organs, as between the gullet and windpipe, between the stomach and colon, or between the contiguous turns of the intestinal canal. Through this unnatural communication, the contents of one part may pass into another, which proves, on some occasions, the cause of instant death, as when the contents of the gullet escape into the windpipe; but, in other instances, life is thus prolonged, as when the pylorus is obstructed, and when the contents of the stomach pass directly into the colon."

Having made these remarks, which are applicable to the various causes of obstruction in the *alimentary canal*, the author proceeds to describe, in the second chapter, the organic diseases of the *gullet*, *stomach*, *and intestines*. This chapter is divided into six sections, the first of which treats "*of Extraneous Bodies lodged within different Parts of the Alimentary Canal, and of their Effects.*"

In this section are collected many extraordinary instances of substances swallowed, with the effects produced, and dissections of patients who died from this cause. The two following shew, very forcibly, how far the alimentary canal can accommodate itself to such intrusions.

"A child, two years of age, swallowed a glass ball, three inches in circumference; it passed through the canal in two days, and did not produce a bad symptom. A female child, three years old, swallowed a pair of compasses, two inches and a half long, and which passed through the alimentary canal in three days, without creating, at the time, or afterwards, a bad symptom."

Section 2.—"*Of Obstructions in the Alimentary Canal, occasioned by Alvine Concretions.*"

In this section is given a more extensive history of human alvine concretions, than is elsewhere to be met with; the materials for which have been principally furnished from the extensive collection of these substances made by Professor Monro, senior: four plates are annexed, which considerably elucidate the subject.

The

The symptoms indicating the existence of these concretions are stated to be irritation, functions of the stomach and intestines, impaired and altered, much griping and sometimes acute pain.

"The pain in the bowels, in some cases fixed to one part, is much more severe upon one occasion than another, especially after taking acids, or food of difficult digestion, and is frequently attended by nausea and vomiting.

"Some patients are much constipated for two or three days, and have yet a constant inclination to go to stool. Others have watery stools, and discharge, along with these, a quantity of a viscid ropy mucus, or blood; after which they are much relieved.

"Some patients discharge their stools involuntarily.

"Upon relaxing the parieties of the abdomen, a very hard, painful, globular, tumor, may generally be felt, most frequently in the course of the large intestines. It can seldom be made to change its plan within the intestine; but often appears to do so in consequence of the change of place of the intestine which contains it. Hence the change appears greatest when the concretion is within the small intestines or arch of the colon, which, from the length of the mesentery or mesocolon, are very moveable.

"The digestive powers being much impaired, the patient becomes very weak and much emaciated; and, from the continuance of the disease, is reduced to a skeleton.

"The pulse, in the earlier stages of the disease, is but little affected.

"Upon the alvine concretion changing its place, and passing down into the sigmoid flexure of the colon, or into the rectum, it creates excruciating torture in the region of the pelvis, and the bowels become much distended, from the passage being suddenly interrupted, and the patient apprehends instant death."

Several cases are subjoined illustrative of the phenomena, management, and termination, of this species of disease of the intestinal canal; and, from his view of the subject, the Professor thinks he has established the following propositions:

"1. That the greater number of *Alvine Concretions* are made up of fibres, which are intimately matted together, and which probably have been attracted by a central nucleus.

"2. That A. C. occasion a derangement of the functions of the alimentary canal, and create griping, obstinate, and long-continued, colicky pains, which are generally limited to that part of the intestinal canal which contains the concretion, and which are occasionally more severe upon the patient taking acids, or food of difficult digestion.

"3. That A. C. may generally be felt within the intestines, and that when two or more of these, are lodged within the intestines, these may be made to strike against each other.

"4. That A. C. frequently change their situation, and pass down into the rectum, which is thereby much extended, and, when so situated, tuated, occasion acute pain and sense of weight in the back part of the pelvis, which is attended by a constant desire to go to stool, which the patient cannot gratify; and they may, by a finger, or by an instrument, introduced into the rectum, be felt within it.

"5. That A. C., formed within the human alimentary canal, are, in some cases, discharged by vomiting, or along with the fœces.

"6. That an A. C., after a certain time, cannot be moved from one portion of the alimentary canal to another, owing to its increase in bulk, to the expansion of the coats in that part which contains the concretion into a *sac*, and to the unnatural constriction immediately below the seat of the Alvine Concretion.

"7. That A. C. must prove a mechanical obstruction to the passage of the aliment through the intestines; and, if proper means be not taken to remove the cause of the obstruction, inflammation follows, which proves fatal.

"8. From the chemical analysis of the concretions, it is evident that Alvine Concretions are of a very peculiar nature, and different from all vegetable and animal productions; and that solvents, sufficiently powerful to act upon those concretions within the intestines, cannot be employed, with impunity, to give the least hope of decomposing those substances within the body, and evacuating them in the ordinary way.

"9. That, in the earlier stages of the disease, while the concretion may be moved from one part of the intestines to another, all that can be done, is to operate on the bowels, partly through the medium of mechanical action, and partly by lubricating the alimentary canal by the exhibition of proper medicines, in order that the concretion may be discharged along with the fœces, or may descend into the rectum, from which it may be artificially extracted.

"10. That after the disease has been of long standing, and when a sac has been formed, which retains the concretion in a certain place, it cannot be removed, should it be lodged within the colon, but by an incision."

Dr. Monro, sen. had suggested that the incision mentioned in proposition x. might, under certain circumstances, be employed with a fair chance of saving life; and a case is given in this volume (page 58), in which, it is probable, such an operation would have been successful.

Section 3.—"*Of Calculi of the Tonsils*."

Of this very uncommon disease, two cases are inserted. Of the calculi, three in number, in these cases, a description is given by Professor Jameson, and a chemical analysis by Dr. Thomson.

Section 4.—Treats of the "*Effects of Arsenic*."

In this section are contained several experiments made on animals with arsenic in its metallic state, and with the artificial sulphurets of this substance. From these experiments it appeared "that the metallic arsenic might be taken by dogs with impunity, to the extent of several grains." It produced produced, however, in these cases, some disturbance in the first passages, and its exhibition was followed by a considerable flow of urine.

Section 5.—" *Of the Effects of Opium.*"

It might be expected that the medium through which opium acts on the system would not now be doubtful; the contrary, however, is the fact. The late Dr. Whytt asserted, that this substance affected the system by the medium of the nerves only. That experimental physiologist Fontana, was equally positive that the blood was the medium. " My father's numerous experiments," says our author, " led him to a different conclusion. He supposes, that opium not only affects the nerves, to which it is primarily applied, but is also absorbed; and, being then mixed with the blood, proves fatal, by its sedative powers upon the nerves of the heart and blood-vessels, and the whole nervous system."

When opium has been swallowed in a deleterious quantity, two distinct sets of symptoms arise. These are " vertigo, torpor, slow and full pulse; breathing at first quick, difficult, and stertorous, and it gradually becomes slower, so that there is a distinct intermission between the inspirations. The pulse gradually becomes more feeble and slower, as the breathing becomes slower. The patient falls into a profound sleep, from which he does not awake, and dies apoplectic, and sometimes is much convulsed. In other cases, vomiting takes place, and the patient becomes afterwards paralytic."

The great object is to remove the poison from the stomach, and this is to be done by emetics. Of those Professor Monro recommends, in preference to all others, sulphate of zinc in solution, in doses of ℈j. to ʒſs. The greater part of the section is employed in describing an instrument for conveying fluids into the stomach.

Section 6.—" *Of the Effects of concentrated Mineral Acids.*"

Of this section, of half a page, it is only necessary to give the title.

CHAPTER III. CLASS 2.

" *Of the Organic Diseases of the Coats of the Intestinal Canal.*"

This chapter is of great importance, as it applies to some of the most formidable and fatal diseases incident to the animal frame. In the distribution of the subject of this chapter, Dr. Monro adopts the following arrangement: 1. Organic derangements of the *villous coat* of the pharynx, gullet, stomach, and intestines. 2. Of the *cellular coat*. 3. Of the mucous coat. 4. Of the peritoneal coat.

The organic morbid alterations of the *villous coat* of the pharynx, gullet, stomach, and intestines, are first explained.

(The

(The investigation is confined to that part of the alimentary canal which is included between the pharynx and rectum.) These organic derangements are treated of under the heads inflammation, thickening, ulceration and gangrene, ulceration and erosion from dysentery, milt-like tumor, polypi, sarcomata, watery tumors, fungous tumors, hæmorrhoids, varicose tumors, strictures, where the other coats are not affected, projecting ring of Dr. Baillie, apthæ, small-pox pustule, deposition of cartilage, formation of bone.

The second section of this chapter (the first section contains only a synopsis of its arrangement) treats of inflammation of the villose coat. There are three facts in this section, which, as having come directly under the author's observance, may not be deemed uninteresting to our readers. It has very generally been understood, that redness and turgescence of the neighbouring arteries are essential to inflammation of the intestines. Dr. Monro has seen, however, "the bowels of patients who died with all the symptoms of inflammation, of a sea-green color; which color could not be imputed to putrefaction, as the body was (bodies were) examined a short time after death." In three cases, the author saw the internal membrane of the pharynx thrown into a state of high inflammation, in consequence of the bite of a mad dog. "*The color of this inflammation was very peculiar; it was not a brilliant red, such as that produced by vermilion, but the purple red, such as carmine or lake gives.*" In the body of a woman who died of diabetes, he "observed an appearance in the intestines similar to that occasioned by inflammation. Upon a more accurate examination, the red color was found owing to a *red jelly* effused between the peritoneal and muscular coats of the intestines." In this inflammation of the villous coat, the mucous, instead of being a mild, transparent, viscid, fluid, is rendered opake, white, sometimes ropy, and even frothy.

Section 3.—"*Thickening of the Villous Coat.*"

The villous coat, from repeated inflammation, remains in a thickened, spongy, and irritable, state; and the common occurrence of effusion of coagulable lymph adds very much to this thickening. The general description is illustrated by cases, and the fact rendered more obvious in Plate XIV. In the Museum of Edinburgh there are several specimens of coagulable lymph being thrown into the cavity of the intestine in such quantity as to take the form of the cavity, and, being discharged per anum, were deemed to be worms of a very unusual kind and size.

Section 4.—"*Ulceration and Erosion of the Villous Coat.*"

This state of disease, is often the occasion of establishing preternatural preternatural communications between neighbouring parts, from the adhesion preceding the ulcerative process. "The symptoms which denote ulceration of the villous coat, are, slight hot and cold fits; hectic flushing of the face; pain in the part affected, becoming less acute, and *detumefaction* of the belly; pulse fuller and slower, often irregular." In the progress of the disease, "the patient becomes very thin, and very weak, and also very costive, hot, thirsty, and feverish, and in this state languishes for a few months; at length he is affected by stupor and delirium, and dies completely exhausted."

Section 5.—"*Gangrene.*"

"Inflammation of the villous coat rarely terminates in gangrene." In this section there are many practical remarks, for which we must refer our readers to the work itself. It also contains a detail of the symptoms of hydrophobia, and a minute history of a case; but, why inserted here, we do not fully see; because, in the fauces, larynx, pharynx, œsophagus, stomach, and the whole of the abdominal viscera, there was not the slightest morbid appearance. In a distant part of the volume we find the "symptoms of mortification of the villous coat." P. 151.

Section 6.—"*Of Inflammation, Ulceration, and Erosion, of the Villous Coat from Dysentery.*"

The history and symptoms of dysentery are here detailed from a manuscript of the author's uncle, Dr. Donald Monro. The concluding part of the section we would gladly insert, as containing some general facts respecting the habits of the mucous membrane; but, when we look to the number of pages yet to go over, we are compelled to forbear.

Section 7.—"*Of the Milt-like Tumor of the Mucous Membrane.*"

This tumor, which our author considers as having entirely escaped the attention of pathologists, he describes as having some analogy to the *anomalous tumor* of his grandfather, to the spongoid inflammation of Burns, and the fungous hæmatodes of Hay; and also to that organic disease of the testes, described by Baillie under the head of pulpy testicle.

"I have called it, says Dr. M——, milt-like tumor, as it resembles in color and consistence the milt of many fishes; and have added the words mucous membranes, because it grows only from membranes of that description.

"This species of tumor generally attains so considerable bulk, as to fill and even to distend to an unnatural bulk, the bowel within which it is contained, as I have seen in the case of the bladder of urine; but, in other cases, this tumor grows from a part only of the mucous membranes, lining the bowel.

"This milt-like tumor, in many respects, resembles the milt of fishes; it is of a pale-red color, and it is also nearly of the same consistence, but rather softer, and has an irregular surface, and is covered by a thin membrane, upon which there are a number of vessels filled by red blood.

"This species of tumor very readily falls to pieces, and mixes in part with water, forming a turbid mixture; and is somewhat hardened by being put into strong spirits. It adheres but slightly to the organ from which it grows, by a number of small processes, which insinuate themselves into the villous coat, which has attained an unnatural thickness; and, when the tumor has been detached, the villous coat of the diseased bowel assumes somewhat of a honey-comb appearance, and it is besmeared by several drops of blood, which are derived from the vessels which extended to the tumor being torn.

"The bowel from which such a tumor grows externally, betrays marks of inflammation; there is evidently an unnatural determination of blood to the seat of the disease, the blood-vessels upon the peritoneal coat being not only larger, but also more numerous than in the healthy state.

"There is another peculiarity in the disease, viz. a very remarkable offensive fœtor; and the organ, containing such a tumor, is as much discolored, and emits as fœtid a smell, as the bowel which has been exposed to the air for several days."

An endeavor is made to give the characteristic marks of this tumor which distinguish it from the tumor to which it was compared in the preceding part of the section, accompanied with a colored plate, (No. V.) drawn from a case here related by Mr. C. Anderson, of Leith.

Section 8.—"*Of Polypi.*"

In this section the varieties of polypus are described, under those of the pharynx, the *gullet*, and the stomach, with an annexed plate, (No. VI.) taken from the disease of a patient, whose case is given.

Section 9.—"*Steatomatous Tumors.*"

The author has not seen an example of this organic disease of the villous coat of the alimentary canal, of course nothing satisfactory is said on it.

Section 10.—"*Fungous of the Villous Coat of the Alimentary Canal, and of Fungous Tumors connected with that Membrane.*"

This disease is not of infrequent occurrence; the tumors are generally of a small size, of a very soft consistence, bleed when torn, and are mostly composed of several small lobules. The accompanying symptoms depend much on the situation of the disease. When in the œsophagus, difficult deglution; and, when in the stomach, indigestion, nausea, and rejection of blood by vomiting; when in the intestines, pain, purging, and discharge of blood by the fœces. An etching,

(No. VII.) represents a cluster of fungous tumors, which stretched across the colon, and which, by intercepting the passage, had occasioned considerable distention.

Sections 10 and 11, treat very concisely "*of Hæmorrhoids, and Varicose Tumors.*"

From these sections we can only notice that sometimes varicose veins of the stomach burst, and large quantities of blood are lost; and the historical fact of Copernicus having died of an hæmorrhage from an hæmorrhoidal varix.

Section 13.—"*Stricture occasioned by a transverse Fold of the Villous Coat of the Alimentary Canal.*

In every case of stricture of the œsophagus, stomach, or intestines, which the author has seen, all the coats of the part affected have been constricted, "so that there was an appearance as if a cord had been drawn very tightly around the diseased part of the alimentary canal." Baillie and Home are particularly referred to, but why are not the later and interesting publications of Copeland and White noticed?

Section 14.—"*Of Apthæ,*" and 15, "*Small-pox Pustules within the Alimentary Canal,*" afford no remark that will be at all interesting, except that the pustules of small-pox in the intestines is "an extremely rare" occurrence.

Section 16.—"*Deposition of Cartilage and Bone upon the Villous Coat of the Alimentary Canal.*"

The parts of the canal most subjected to this disease, are the œsophagus, colon, and rectum. When the cartilaginous stricture happens to the rectum, the symptoms are described to be,

First, "a slight difficulty in making water; this is followed by nausea, impaired digestion, colicky pains, tenesmus, and habitual costiveness. The fœces have not their usual size and shape, but resemble small earth-worms, and are expelled only after a considerable effort; and, when the patient is very costive, blood is sometimes discharged with the excrement; and generally a quantity of fœtid mucus, ichorous, or purulent matter. When the disease has been of some duration, solid fœces cannot pass; and the contents of the intestines are discharged in a liquid form, and even these are passed only after a considerable effort."

We now come to the second general division of this chapter, "*Organic Derangements of the Cellular Coat of the Alimentary Canal,*" to which, with an unfortunate anomaly as to the construction of the volume, is continued the term section. According to the synoptic view (p. 111) it contains three subdivisions: phlegmon and its consequences; albuminous tumors; and diseases which originate in the other coverings of the intestines. As we have professed to to follow our author precisely, we must, with him, call this second general division section 17. This section contains general observations on inflammation of the cellular coat, and its consequences, abscess, &c. Section 18, is on a subject of considerable novelty and interest, "*the Deposition of albuminous Matter in the cellular Coat of the Alimentary Canal.*

"This organic derangement is of a very peculiar nature, and very generally affects at the same time different bowels of the abdomen and pelvis, and also the lymphatic glands in the vicinity.

"In the earlier part of the disease, we observe small hard tumors, about the size of a pea, in the cellular substance, which grow inwardly, and which push before them the villous coat.

"In the more advanced stages, the diseased bowel attains an unnatural hardness and size, and its coats are prodigiously thickened in some places. Tumors of a pyramidal figure grow inwards from the thickened parieties, by which the diameter of the affected part is much lessened. Upon making a section of the coats, we observe the peritoneal coat somewhat harder, whiter, and thicker, than common, but no appearance of the cellular and muscular coats, for their place is occupied by the albuminous substance."

The liver, testes, uterus, and lymphatic glands, are all subject to this disease, and have their texture and appearance exceedingly changed by it; as it appears in the liver, it is here the subject of a particular description.

The third subdivision has no more than its title.

The third general division, "*Organic derangements of the muscular Coat of the Alimentary Canal,*" making the 19th section, contains inflammation and its consequences, spasm, and palsy. The contraction of the middle of the stomach, as stated by Mr. Home, is admitted to be nearly correct; and, in the part appropriated to spasmodic contractions of the intestines, the species or varieties of colic, in nosologists, are slightly treated of. From this part we extract the following passage, on the possibility of ascertaining the seat of the contraction by the symptoms.

"Pain in the navel has been supposed to be characteristic of contraction in the *jejunum or ilium;* nervous oppression, torpor, inclination to sleep, when the *stomach is empty;* occasional distention of the abdomen, pain in the right side, which is occasionally very great, and which stretches to the back, and occasionally to the top, of the right shoulder, and which changes its place upon the expulsion of air, slight yellowness of the eyes and countenance, with an irregular and soft pulse, that of the *duodenum;* and pain in the right side, stretching toward the region of the liver, that of the *caput cæcum coli.* Acute twisting pain about the navel, which is not increased on pressure, the dragging inwards of the parieties of the abdomen, which, when pressed pressed, feel hard and knotty, tenesmus, and obstinate costiveness, are pathagnomonic symptoms of the painter's colic."

The muscles of the palsied extremities, consequent on this species of colic, not only lose their natural size, but have their structure converted into a suety substance; as has also happened in rachitis, in scrofula, and in that disease called osteosarcoma.

The 20th section, still subjected to the anomaly before noticed, constitutes the fourth general division of this chapter, and comprehends the "*Organic Derangements of the Peritoneal Coat.*" The subdivision of this treats of inflammation—Section 21, small tumors growing from the peritoneal coat—Section 22, ossification of the peritoneum—Section 23, hydatids.

The last of these subdivisions, under the denomination of section 23, treats very fully on the obscure form of animal life in the hydatid; and from which the following corollaries are deduced:

"1st. That hydatids are not peculiar to any one part of the human body, and (but) are most commonly connected with the investing membranes of the liver, ovaria, or kidney.

"2d. That there is no resemblance between the hydatids which are peculiar to quadrupeds, and those of the human body, as is obvious, by comparing the preceding description of the hydatid of the human body, with those of the hydatids of quadrupeds, which have been published by Hartmannus, Tyson, Pallas, Schroeder, Fontana, and E. Horne.

"3d. That there is every reason to conclude that hydatids are animals.

"4th. That observation and experiment have not yet determined in what manner hydatids are generated, or deposited, within certain bowels.

"5th. That, as the smaller hydatids adhere to the inner surface of the larger, the larger hydatids may be called pregnant; or that these animals are multiplied, like some vegetables, by the adhesion of the smaller hydatids to the coats of the larger hydatids.

"6th. That the coats of the bowels containing the hydatids are much more frequently destroyed, than when water only has been collected within them; hence the hydatids escape from their original situation, and sometimes find their way by unnatural passages into the intestines, urinary or biliary canals, windpipe, &c.

"7th. That many patients recover upon the discharge of the hydatids.

"8th. That hydatids may, even when adhering to one of the bowels of the abdomen, be removed by incision, provided there exists an adhesion between that viscus and the parietes of the abdomen."

The fifth general division, called section 24, treats of "*Organic*

"*Organic Derangements of all the Coats of the Alimentary Canal.*"

Under this division are investigated the various degrees and situations of stricture of the alimentary canal, scrofula of the intestines, coats of the alimentary canal reduced to a pulpy state, induration of the coats of the intestines, dilatation and rupture of a part of the alimentary canal, organic diseases of the mucous glands of the alimentary canal, scirrhus and cancer of the gullet, stomach, pylorus, and intestines, scirrhus rectum, enlargement of the mucous glands of the alimentary canal.

Three plates accompany this part—No. 8, representing a fatal stricture of the œsophagus; No. 9, a cancer of the œsophagus; and No. 10, stricture occasioned by cancerous tumors at the cardia, and cancer of the stomach.

The third CHAPTER treats of those obstructions which "*originate from a Displacement of a Portion of the Alimentary Canal.*" These causes of obstruction are *Intus-susceptio*, *Procidentia Ani*, and *Hernia.*

Section 1.—"*Intus-susceptio.*"

This derangement occurs much more frequently in infancy than in advanced life. It is of "two very distinct kinds, that which is unattended by inflammation, and that attended by acute inflammation and its consequences. The former, which occurs generally during infancy, in most cases does not merit the name of a disease, as it does not derange the functions of the alimentary canal; whereas the latter, which may be ranked among the diseases of manhood and old age, is one of the most acute and fatal disorders incident to humanity."

The phenomena that distinguish the intus-susceptio, accompanied with acute symptoms, from inflammation of the intestines excited by other causes, are stated to be,

"The sudden appearance of the symptoms after violent straining at stool, the impossibility of throwing up by the anus as much liquid as in a state of health, together with the sudden appearance of a hard tumor on the left side of the abdomen, and which is painful on pressure."

The progress of the disease is well marked in a case from Professor Monro, sen. with a plate, No 21.

Connected with this derangement of the intestinal canal, is the discharge of a portion of intestine by stool. Alarming as this appears, it is not always dangerous. An instance is here given of fifteen inches of the ilium being brought away by stool, and the patient having good health for many years after.

Section

Section 2, "*Procidentia Ani,*" is so connected with section 1, that it is barely mentioned here.

Section 3, "of *Hernia,*" is important and extensive, occupying the volume from page 363 to 542; and is illustrated by several plates.

To give such an analysis of this section as would be at all satisfactory to our readers, would occupy more space than can be allowed to it; we must therefore refer to the work itself, and only observe, that the author has brought together much curious matter both anatomical and chirurgical, on every species and variety of the disease.

CHAPTER IV. "*Of Mal-formations of the lower Part of the Alimentary Canal,*" is confined to a description of malconformations of the rectum, under eight varieties.

1st, and most common, is that in which the rectum is covered (when it should terminate in the anus) by the common teguments, or by a membrane of considerable thickness.

2d, When the membrane which obstructs the rectum is internal.

3d, Unnatural contraction of the rectum.

4th, Where the rectum terminates in the bladder of urine, urethra, vagina, or womb.

5th, The rectum terminating in the vagina, through which the fœces are discharged.

6th, Where the rectum is sometimes entirely *awanting* (wanting).

7th, Where the rectum opens through the os sacrum.

8th, Where the rectum has been continued through the vagina, and has terminated external to the vulva.

CHAPTER V. "*On the Worms which infest the human Alimentary Canal,*" gives a short detail of the history and anatomical structure of the *Tenia solium*, *T. dentata*, *T. lata;* the *Ascaris vermicularis*, *A. lumbricoides;* and the *Trichuris hominis.*

Of the last, the *Trichuris hominis*, as being a more rare species than the former, we shall insert the professor's description.

"The body is about an inch long, and it has a filiform tail, about an inch and a half in length.

"Different authors vary in their opinions respecting the anatomy of this worm. According to some, the animal has a proboscis, which it can eject at pleasure; according to Goeze, that is the penis of the animal.

"The stomach and intestines form a long canal, which proceeds from the head to the extremity of the worm, and is largest at the beginning; is much smaller at the tail of the animal.

"The ovarium, which frequently contains ovula, and a limpid fluid, fluid, is a convoluted canal, and similar to that of the female Ascaris vermicularis."

We had been made to understand, that after chapter five there was to be a sixth, to consist "of an enumeration of the causes which lead to an enlargement of those neighboring organs, which by pressing upon the alimentary canal, prove a mechanical obstruction to the progress of its contents." But we have not found it in its promised place.

Upon the importance of the subject of this volume there will be but one opinion—upon the quality of the materials with which it is formed, there will necessarily exist diversity of estimation—upon the style in which it is written, and upon the employment of low and popular terms in the place of those consecrated to science, we have been free in our animadversions. But, if we should be thought to have fallen into any semblance of severity, we deprecate the charge. Our duty to the profession, and to ourselves, required that we should speak out; and this we have done in the spirit of perfect charity with Professor Monro. When we see men in high stations negligent of appearances in their public acts, we feel for the credit of themselves and of the profession to which they belong; and we tremble for the effect of precedent. Among physicians we may still find correct, energetic, and elegant, writers: the Augustan age of medical literature has not yet passed away; we cannot, therefore, permit the example of this volume, the chance of debasing the style of medical compositions, without an effort to avert the evil.

---

*Practical Remarks on Insanity, to which is added, a Commentary on the Dissection of the Brains of Maniacs; with some Account of Diseases incident to the Insane. By* Bryan Crowther, Member of the Royal College of Surgeons in London, and Surgeon to Bridewell and Bethlem Hospitals. 8vo. pp. 130. Underwood; London, 1811.

As far as opportunity and situation can contribute to enable a man to obtain knowledge in a particular and interesting branch of the profession, Mr. Crowther is eminently favored. If we are disappointed in the quantum and quality of information which has been the result of those favorable circumstances, perhaps we raised our expectations too high. Our chief view, however, in analysing medical publications, being to extract whatever is curious and useful, we shall endeavor to select the most valuable parts of this treatise, and enable our readers to decide upon the author's pretensions.

The first section treats of the causes of insanity, and the author candidly states that he is very ignorant of them: the concluding paragraph contains the sum of his information on that head.

"As to the general cause of insanity (observes Mr. C.) I know nothing; and my ignorance is less to be regretted, as the celebrated Cullen declares, that, as 'we know there have been many instances of insanity from which the persons have entirely recovered, it is difficult to suppose that any organic lesions of the brain had taken place.' Thus much I advance in support of the general cause of madness, not having its origin in an altered texture of this organ; and, as to those who are incurably insane, it matters not whether they possess one condition of the brain or the other, and whether it be the cerebrum aridum of Morgagni, or the mania corporea of Dr. Cullen." P. 21.

The appearances of the brain and its membranes on dissection, are dispatched in a short section. They are stated to consist "in opacity of the arachnoid membrane, which was sometimes occasionally thickened; a preternatural determination of blood to the membranes as well as the brain; together with an effusion of water between its membranes, its convolutions, and into the ventricles." In some instances ossification of the arteries occurred, and the pineal gland was charged with sabulous matter; but all these appearances frequently exist when there has been no insanity. Mr. Crowther therefore regards them as the effects rather than as the causes of mental derangement. He states (we much regret he has not mentioned his authority) that

"One anatomical teacher of acknowledged eminence entertained the opinion that the brains of maniacal persons were always morbidly affected; but, after having examined a number of additional cases, and not observing in many heads any vestige of actual disease, he was led to the conclusion that the cause of insanity did not consist in diseased affection of the brain.

"Another gentleman, whose anatomical skill is also acknowledged, inspected, in Bethlem hospital, several heads at my request, some of which he declared, had they been examined elsewhere, he should have pronounced to have had no unusual appearance." P. 26.

Mr. Crowther has taken some pains to investigate the effect of local diseases in cases of vesania. From the result of his experience, it seems that surgical complaints have no influence on mental derangement. It has been supposed by some individuals, that insanity secured the patient from the attacks of epidemics; whilst others have asserted that these complaints not only affected maniacs, but tended to their cure. The following table affords only scanty evidence, but sufficient, in our opinion, to set aside the first supposition, and to render the second doubtful. From 1783 to

1793,

1793, twenty-seven patients took the natural small-pox in Bethlem hospital. Of these, 18 died; one was discharged sick and weak; five recovered from the small-pox, and were cured of their insanity; three recovered from the small-pox, but remained incurably mad. The deaths of course cannot be admitted as evidence on either side the question. We must therefore look at the number of recoveries from mania after small-pox, which we find was in the proportion of 5 to 3; but this, Mr. Crowther observes, forms "nearly the aggregate of mad persons who recover without the intervention of this malady" (small-pox). "It appears, from the books of Bethlem hospital, that not quite a half, but more than one third, of its patients are discharged cured of their insanity." P. 51.

The facts at present before us certainly are insufficient to determine the question: we have merely concurred with our author's opinion, founded on the evidence which he has adduced, and by no means presume to draw any general conclusion from such partial instances. The subject is curious, and we should be glad to receive information upon it. The following case, in which small-pox seemed to produce a favorable effect, as far as a solitary case can have weight, deserves consideration.

"W. K. an incurable patient (in Bethlem) took the infection: he was conveyed to the Small-pox hospital, July 16th, 1794, and returned to the charity August 14th following, well of the small-pox, and restored to his senses. This person's case is the more worthy of attention, as he was on the incurable establishment, and that his violence of demeanor was such, that it became necessary to chain him to his apartment."

He continued well about eight months, and was discharged; but in a fortnight was again brought to the hospital in a state of insanity, which was attributed to irregularity and intemperance during his short absence.

In the three succeeding sections the author treats of mortification of the feet, and nates, and sphacelus of the toes; but it does not appear that these affections in maniacs require a different treatment from that which is usually pursued when they occur in other cases.

The section on management contains little novelty, as Mr. Crowther observes "the tale I may change, but the means of restraint are the same, limited to the strait waistcoat and confinement to the room." Many instances of insane people being convinced of their error by reasoning with them, have been quoted by writers; we shall state two related by the present author.

"A man, who conceived himself to be the Father, Son, and Holy Ghost,

Ghost, asked me (Mr. Crowther) what were my notions of the doctrine of the Trinity? I answered him, 'it is a mystery; and it would cease to be such, if you or I could comprehend it.' Satisfied with such answer, he made no reply, nor ever after mentioned the subject.

"Another patient imagined himself to be Jesus Christ; and, in proof of it, shewed me a scar he had in his side, which he said had been occasioned by his having been pierced with a spear. I remonstrated with him on his assertion, and remarked, that our Saviour was wounded on the side opposite to that he had indicated as the part wounded in himself. Convinced, and apparently ashamed, at the consciousness of the fallacy of his own reasoning, the patient recoiled, hid himself under the bed-clothes, and never reverted to the impression under which he had previously labored." P. 94.

The section on the medical treatment of the insane, is chiefly extracted from preceding writers. In undertaking this task, the author states his "principal view has been to collect and concentrate the practical ideas of others in such a manner as will render more useful this tract, upon this intricate but interesting subject."

We conceive we have now given the cream of Mr. Crowther's publication. Should our readers, however, be of a different opinion, we recommend them to purchase the volume.

---

*A Companion to Mr. Bullock's London Museum and Pantherion, &c. &c. &c. By* William Bullock, F.L.S. and H.M.D.S. 12mo. London, 1812. pp. 136–57.

We have often had occasion to notice the industry of Mr. Bullock in collecting, and the taste displayed in arranging, his immense stores of natural history; and which he has now deposited in a building erected for their reception, under the denomination of the *London Museum.*

The visitors to this place of amusement and rational gratification had to regret that they often left it with little information or distinct knowledge of what they had seen and cursorily admired; because a descriptive guide or catalogue raisonnée, which might direct them through this wilderness of the beauties of nature, and bring them acquainted with the established facts, or rational conjectures that applied to the individual specimens, was wanting. This *desideratum* is in a measure supplied by the little work now before us: it, however, by no means reaches our idea of what such a catalogue might be made; and, though in its present state exceedingly useful to those whose curiosity leads them to this depository, it might go much further, and become a directory to the whole system of created things, the specimens of which, found here, would illustrate the text text in a way infinitely superior to the most finished engravings. With this view, every specimen should have, at least, its generic and specific name, and its native place, with a reference to the best authorities. When the specimen was new, or non-descript (of this kind we meet with many), all the facts respecting it which have come to the knowledge of the ingenious collector, should be scrupulously given. The Linnæan arrangement, for we have no idea of any that can excel it, in the extended view we have suggested, should be continued; the species wanting to fill up the genera might be noticed (in a foot note), as affording a probable means of acquiring those species; and we see no reason why, in a place like London, secure from the desolations of war, commanding wealth beyond calculation, and holding an intercourse with the whole habitable earth, the plan of Mr. Bullock might not be pushed on to a complete collection of Natural History. We should be glad to see, in addition to this, a library and extensive *Herbaria.* We acknowledge this *coup d'œil* to be vast, but the powers of man, bent to one object, and supported by public approbation, are not to be estimated. "All the performances of human art, at which we look with praise or wonder, are instances of the resistless force of perseverance; it is by this that the quarry becomes a pyramid, and that distant countries are united by canals. If a man was to compare the effect of a single stroke of the pick-axe, or of one impression of the spade, with the general design and last result, he would be overwhelmed by this sense of their disproportion; yet those petty operations, incessantly continued, in time surmount the greatest difficulties, and mountains are levelled, and oceans bounded, by the slender force of human beings."

It is not consonant to our plan to go into a minute investigation of this interesting subject; and it would be superfluous to give a regular analysis of what in itself is only an analysis; but we shall not dismiss it without doing the proprietor of the *London Museum* the justice of saying, that his collections in various classes of the *systema naturæ* are extensive, choice, and valuable. In the class of birds we have been particularly attracted by the singular and beautiful genus *Paradisea*, the species of which here deposited are numerous, and many of them rare. Of the 48 species of the genus *Cuculus*, we find 40 here. But of the genus *Trochilus* (humming bird) this museum may boast, for it contains 100 species, many of which are non-descripts. The species of the classes Amphibia, Insecta, and Pisces, are also very numerous; and the collection of shells, corals, Madrepores, and minerals, respectable.

The

The latter part of this volume contains the description of a novel exhibition, so far it regards the distribution of the subjects, under the title of PANTHERION. It consists of a large collection of animals distributed throughout a tropical forest, intended to represent them as nearly as possible, in a state of unrestrained nature. The forest itself is an object of some interest, as it is formed of trees and plants of the torrid zone, modelled from nature, or the best authorities. Among them we observed *Atrocarpus incisi*, *Agave Americana*, *Musa sapientum*, *Passiflora quadrangularis*, *Dimocarpus litchi*, *Annona reticularis*, *Quercus suber*, *Carica papaya*, *Psidium pyreferum*, *Borassus fabelliformis*, *Urania speciosa*, *Cocus mucefora*, &c. &c. &c.

We can recommend this little work to all those who visit the *London Museum;* and, should its author ever think proper to adopt the hints we have given him, it may become a useful and interesting addition to the library of the naturalist.*

* An Inquirer in our last number (page 380) will see, by the above, what assistance he may expect in his visits to Mr. Bullock's Museum.

